# The Globalisation of Cardiology and Cardiovascular Diseases in the World–Society—A Case Study with a Special Focus on Heart Failure

**DOI:** 10.3390/ijerph17093150

**Published:** 2020-04-30

**Authors:** Benjamin Quasinowski, Tao Liu

**Affiliations:** 1 Institute of Sociology, University of Duisburg-Essen, 47057 Duisburg, Germany; 2 School of Public Affairs, Zhejiang University, Hangzhou 310058, China

**Keywords:** world–society, globalisation, global health, world–polity, cardiology, heart failure, neoinstitutionalism

## Abstract

While there has been a shift of attention in global health towards non-communicable diseases, we still know little about the social mechanisms that have allowed these diseases to emerge as topics of global concern. We employ a sociological approach to globalisation in order to reconstruct how cardiology, with our special focus being on heart failure research, has become global, and thereby placed cardiovascular diseases on the agenda of global health. Following sociological theories of world–society and world–polity, we identify a number of preconditions that had to be met so that the globalisation of cardiology could set in. Amongst them were technological innovations, the emergence of an organisational infrastructure on the national level, the appearance of cardiological journals, and an internationally standardised nomenclature. More recently, new drugs and treatment strategies, new specialist journals, and new international standards allowed the subspeciality of heart failure to globalise. Our findings are based on the history and sociology of cardiology, and on our analysis of a broad range of other documents, including scientific articles, guidelines, and policy documents. Additionally, our analysis included two datasets, one containing information on national cardiac societies, and the other containing data on publication output in cardiology.

## 1. Introduction

### 1.1. Background and Research Question

Over the twentieth century, more and more diseases attained the status of global threats and risks. In the case of infectious diseases, the pathways of their globalisation seem natural: new linkages between far-away regions of the globe have been established, the movements of people have substantially intensified, and so, diseases have moved along these pathways [1]. Aside from the current concern with the globalisation of SARS-CoV-2, the Spanish flu is still the most notorious in a range of examples of the globalisation of diseases and health risks. However, around the same time that the Spanish flu terrified populations across the globe, new international health organisations, most importantly the Health Organisation of the League of Nations, were making headway on creating a global framework for health policies and politics. Eventually, this framework made it possible to not only perceive health and diseases as issues of worldwide concern, but also to take coordinated action on a global scale [2]. The efforts around the eradication of smallpox, eventually succeeding in 1977, serve as the paradigmatic case of successful coordination of global health action. Throughout the past century, it seems, global health concerns have predominantly been with infectious diseases.

Nevertheless, in more recent years there have been indications that the paradigms of global health have somewhat been taking a new turn. Non-communicable diseases (NCDs)—including, first of all, diabetes, cancer, respiratory, and cardiovascular diseases (CVDs)—have lately become much more prominent on the agendas of global health [3]. On the one hand, their upgraded status seems completely justified, as more than seventy percent of worldwide deaths are due to NCDs in general, and, for instance, just over thirty percent of worldwide deaths are due to CVDs [4]. That is, from an epidemiological perspective, CVDs are globally on the rise.

Nonetheless, currently, we do not actually know why and how the tide has been turning. Disease–deterministic explanations that see the new emphasis on NCDs in global health as simply resulting from the worldwide prevalence of these diseases fall short, since, arguably, many diseases had existed worldwide long before they emerged as issues of concern on the agendas of global health. Moreover, while certain serious diseases affecting populations all over the world may linger in silence and attract little attention from global health planners, certain other diseases, even in spite of their relative harmlessness, suddenly come to the fore and result in substantial efforts of globally coordinated action.

An alternative approach for understanding why and how certain diseases and health risks become issues of global concern allocates more attention to the institutional underpinnings that facilitate their emergence on the agendas of global health. Certainly, once a medical discipline has been stabilised on a global level, it is much more likely that particular discourses and objects of knowledge associated with that discipline are able to attract attention from global health circles. That is, although the prevalence of a particular disease may be increasing, it still takes specific conditions, such as a differentiated institutional infrastructure, so that communication about the rising prevalence of that disease can eventually result in the widespread perception of that disease as a global health concern. Therefore, it is important to ask just how medical disciplines become global, and how they connect to global health policies. Sociological theories of globalisation, based on empirical research, can be useful tools to tackle these questions. Therefore, from a sociological perspective, which complements the epidemiological perspective, we may ask how and why CVDs have risen to global relevance.

### 1.2. Purpose of This Study

In this article, with the case of cardiology and its subspeciality of heart failure research, we demonstrate how an institutional framework has successfully been installed, and then helped cardiovascular diseases, amongst them the syndrome of heart failure, to become issues of global concern. Our main argument is that certain preconditions had to be met before cardiology, and later heart failure research, could globalise. These preconditions included technological innovations, the emergence of a unitary nomenclature, and, perhaps most importantly, the expansion of cardiac societies and scientific journals worldwide. Once these preconditions were met and cardiology, along with many of its subfields, such as heart failure research, globalised, particular cardiovascular risks and diseases could easily become topics of global health.

We show how cardiology has become a global discipline throughout the past century and how as such it has gained considerable influence on the shape of global health as a policy field. Additionally, with global cardiology as the backdrop, we inquire into the specific preconditions that were necessary for the subspeciality of heart failure to gain sufficient momentum in order to rise above the limits of its local contexts of origin, eventually leading towards its globalisation. For the purpose of this article, our focus on heart failure research as a cardiological subspeciality is instructive. On the one hand, the growing prevalence of heart failure – a complex clinical syndrome that is often resulting from a diverse range of CVDs – has been recognised and it is seen as causing enormous health expenditures worldwide [5]. However, since heart failure has been a serious health problem in many countries for quite some time, the epidemiological recognition of its increasing prevalence alone cannot explain the fact that the syndrome has also been pushed onto the agenda of global health concerns. We argue that the growing global awareness of the syndrome, expressed, for example, in the work and efforts of international and supranational health organisations, such as the World Heart Federation (WHF) and the European Society of Cardiology (ESC), is due to the specific institutional infrastructure that is the focus of this article.

## 2. Theoretical Backgrounds

### 2.1. The Neoinstitutional Approach to Globalisation

The neoinstitutionalists from the Stanford School have developed the sociological ‘world–polity’ theory, which posits an ontological level of global social reality beyond the constraints of methodological and theoretical nationalism. Based on abundant empirical surveys, for decades, the neoinstitutionalists have argued that policies, institutional programmes, and rationalised models in different fields, such as economic development, education, public health, and environmental protection, have spread from the West to other parts of the world. This spread has been possible because new institutional frameworks and norms have proliferated, in spite of the absence of a central global authority, such as a ‘world state’ or ‘world government’. Consequently, since the end of World War II, certain discourses and practices have become dominant, with a claim of universal validity, including the Global North and Global South [6,7,8]. Globally diffusing scripts and models are culturally underpinned by values and norms such as universalism, individualism, rationalism, and general socially progressive ideas, legitimating the behaviours of nation states, organisations, and individuals [9]. Their diffusion has occurred rather in a highly decentralised communicative context that has been institutionally configured by a ‘world–polity’, comprising a set of international agencies, supranational communities, and transnational actors [10]. For instance, UN departments and agencies, including a number of international governmental organisations, figure as ‘objective disinterested others’ [11], who are responsible for transferring UN norms and concepts to member states. The primary methods of global diffusion are not based on the coercive enforcement of policies by a centralistic actor, but rather on the persuasive power of the world–society’s beliefs and values, which are voluntarily taken up by national states, organisations, and individuals [9]. Against this backdrop of a global diffusion of ideas, scripts, and models, a trend of global isomorphism has been identified by the Stanford theorists, suggesting that a global convergent development in various fields has occurred, in spite of national and local socio–cultural disparity [11].

### 2.2. Preconditions of Globalisation

A common feature of much research on globalisation is that it largely argues from a mere theoretical perspective, and takes the globality of its particular object of study for granted, that is, not worthy of being empirically tested. Other research traditions have tried to put the concept of globalisation on a more empirical footing by investigating the extent and/or density of worldwide connections of different kinds [12]. This enables them to reach conclusions concerning the degree of the globality of particular phenomena. These research traditions often understand globalisation through what the sociologists Heintz and Werron refer to as globalisation through interlinkages and connectivity [13]. Heintz and Werron suggest that there is another, complementary mechanism of globalisation, to which they refer to as globalisation through description. According to them, globalisation through description is, to a considerable extent, based on the communication of comparisons [13]. They investigate how the communication of comparisons has triggered globalisation processes in the fields of competitive sports and science, and how initially local horizons of comparisons have become global. They are less interested in describing factual globalisation, but inquire into the preconditions of the possibility of globalisation. A main assumption of their approach is that from the outset, the occurrence of globalisation is highly unlikely, and that a set of very specific preconditions must be fulfilled before the globalisation of particular societal fields can actually set in. The historical reconstruction of individual societal fields’ paths to emerging global orders is seen as a central task of investigations whose research object is globalisation through description. One advantage of making the specific preconditions of globalisation the focus of one’s analysis is that it avoids demanding theoretical presumptions in regard to factual globalisation. Instead, it shifts the analytical focus to the investigation of clearly specified empirical phenomena.

Heintz and Werron argue that public discourses of comparison haven been major drivers of globalisation in a number of societal fields. When a particular societal field begins to transcend the thresholds of its local boundedness, due to the emergence of translocal comparative discourses, according to Heintz and Werron [13], it is likely that prior to that certain historical precondition had been fulfilled. Only then can a global reference frame come into existence. They note that there had been comparative discourses in competitive sports and science for a long time, but up to the second half of the nineteenth century, these discourses had been contained to specific localities, and a global reference frame had not yet come into existence. In other words, although scientists had been engaged in collaborative research, published studies, read scientific ‘letters’, the linkages between the individual localities of these endeavours had been very limited. Those linkages that enabled the emergence of increasingly translocal comparative discourses came into being only in the second half of the nineteenth century. Only then were scientists able to relate their work to a potentially, though not necessarily factually, global reference framework [13].

Abstracting from their empirical cases, Heintz and Werron singled out a number of preconditions that must be fulfilled for globalisation to occur in any societal field. Firstly, there must be a solid basis for the continuous production of public, comparable events. For instance, in the case of science, new research and findings have to be published continuously. Secondly, comparability must be possible beyond the contexts of particular localities. For instance, unitary methods of measurement, and also measurement units, may foster the translocal comparison of research findings. Moreover, such kinds of standardisation are likely to result in a normalised scientific language, and also in specific theories and generalisations. Thirdly, criteria and dimensions must be defined such that individual events (of comparison) can be put into an overarching framework linking the events to be compared with each other. In science, for instance, truth claims are bound to certain principles of argumentation, and research findings are evaluated according to very specific criteria, of which peer-review procedures are an example [13].

According to Heintz and Werron, in the sciences, the preconditions of globalisation had been largely met at the beginning of the twentieth century. Over the second half of the nineteenth century, new measures and methods of measurement had been introduced to secure the validity of experiments and observations. Scientific journals had begun to appear in a wide range of countries and disciplines. Scientific language itself had become more and more standardised, in accordance with internationally emerging norms of science. Consequently, participants from geographically far apart and culturally diverse regions were able to communicate about their research. Once research findings could be communicated beyond a particular locality, they could be taken up, be supported, circulated, critiqued, and, perhaps most importantly, be fed into the already existing citation and research networks. Moreover, as awareness amongst scientists of their individual positions as members of a potentially worldwide scientific community had steadily been on the increase, a worldwide communicative horizon had emerged, to which each member could—and indeed had to—relate his or her work. For scientists it had become more and more important that any member of their specific ‘imagined community’ [14] could potentially become a recipient of her work. This had to be taken into account when one intended to communicate the results of one’s research, and, of course, such considerations found institutionalised repercussions, e.g., in the structures and genres of scientific literature.

Heintz and Werron investigated the preconditions that allowed globalisation to set in in a number of different societal fields. However, what remains an issue is whether the globalisation of a particular field necessarily implies the globalisation of its subfields. Moreover, if the preconditions for globalisation are met in a particular societal field, does this imply that the conditions for globalisation are also met in its subfields? On the one hand, we may take for granted that medicine, along with some of its disciplines, such as oncology or cardiology, are in fact global fields today. However, we still need to closely scrutinise the globalisation of, for example, an emerging subspeciality such as heart failure research.

## 3. Materials and Methods 

The theoretical approaches just sketched, determined the methods and materials used in our own study to a considerable extent. Heintz and Werron’s approach, applied to the fields of science and sports, respectively, was historical. They had access to an extensive secondary literature that takes a social science or humanities perspective on the development of these fields. In regard to the field of heart failure research, the amount of available secondary literature is extremely scarce and scattered. Where possible, we followed existing forays and tried to apply them to our specific questions. But we had also to do our own research and work with original sources, such as scientific articles and clinical practice guidelines. In contrast to the situation with the secondary literature on the particular field of heart failure, there is a considerable amount of secondary literature on cardiology as a medical discipline. Since heart failure research developed from within cardiology, this literature served as a backdrop for our analysis of the emergence of a global field of heart failure research. Additionally, we interviewed several experts in the field of heart failure from Europe, the United States, China, India, and several other countries. Insights from these interviews allowed us to contextualise our document analysis, and also allowed us to clarify specific technical questions that had emerged over the course of our study.

In order to understand how cardiology has been institutionalised across different countries of the world, we created a dataset that includes the years of foundation of a relatively large number of national cardiac societies. The webpage of the European Society of Cardiology (ESC), which contains a section with data on the ESC national cardiac societies, and a section with data on affiliated cardiac societies, was the primary resource for this dataset [15]. In addition to the data found on the ESC’s webpage, we added data on the American Heart Association and the American College of Cardiology. In total, our dataset comprises data on 99 national cardiac societies. Although it does not contain data on all national cardiac organisations of the world, it is comprehensive, and probably covers a very significant part of the total number of national cardiac societies worldwide.

Furthermore, since our theoretical approach predicts that the existence of scientific (specialist) journals is an important factor enabling the translocal communication of comparisons, we created a second dataset that contains data on cardiological journals. It is based on data provided by SCImago [16] and was compiled from the SCImago subject category ‘Cardiology and Cardiovascular Medicine’. A deficit of this dataset is that it does not contain information concerning certain cardiological journals. For instance, Zeitschrift für Kardiologie, a German journal founded already in 1909, is not included in the SCImago dataset under this name. We suspect that the SCImago dataset particularly misses journals that are published in languages other than English. Nevertheless, since SCImago offers one of the most comprehensive datasets publicly available, we have based our analysis on this dataset.

The SCImago dataset comprises 358 journals. For each journal, it contains such information as the journal’s SCImago Journal Rank, H-index, publisher, country of publication, and more. For our current purposes, we were particularly interested in each journal’s year of foundation. SCImago does not provide information on that directly. However, it provides information on years of coverage. We took each journal’s first year of coverage as indicating the journal’s year of foundation. In many cases, this proved to be a correct guess and the first year of coverage was indeed equivalent with the journal’s year of foundation. In most other cases, the first year of coverage was quite close to the year of foundation. Thus, for the purpose of our study, we inferred the year of foundation of a journal from the first year of coverage shown in the SCImago dataset.

Furthermore, we added a variable to categorise each journal’s scope as either being ‘general’, i.e., covering the discipline of cardiology in general, or being ‘specialist’, i.e., covering a specialist field within cardiology (e.g., heart failure, imaging methods, atherosclerosis, electrophysiology, cardiac surgery, cardiovascular nursing, pediatric cardiology, or translational science). Our categorisation was based on the journals’ titles and the brief descriptions (often entitled Aims and Scope) that can be found on the homepage of most journals.

Finally, in order to put the publication output in the field of heart failure research in perspective, we conducted a search on Web of Science [17], and compared the number of publications in the field of heart failure with the number of publications in cardiology in general (the search was conducted on February 17, 2020). The search for literature in cardiology in general, with the query string ‘SU = “Cardiovascular System & Cardiology”’, returned 1,603,129 records. We then conducted a second search within a subset of the results of the first search, with the query string ‘SU = “Cardiovascular System & Cardiology” AND (TS = “heart failure” OR TS = “cardiac failure”)’. This search returned 134,321 records. Annual publication outputs were then mapped on a timeline from 1910 to 2018.

## 4. The Global Field of Cardiology

The heart has been a ‘knowledge object’ [18] since ancient times. However, William Harvey’s discovery of the blood circulation, and, thereafter, an emerging mechanistic understanding of the heart in terms of a pump, were preconditions for integrating it into the modern biomedical perspective. There had been struggles between proponents of mechanistic and vitalistic views, but a mechanistic view has eventually been prevailing since the second half of the nineteenth century [19]. Not surprisingly, the success of the mechanistic over the vitalistic view has coincided with massive processes of differentiation within medicine, starting in a number of industrialising countries in the nineteenth century [20]. It has been argued that this differentiation of medicine has correlated with an ever more differentiating and, literally, dissecting medical gaze, which itself resulted from the institutionalisation of anatomical pathology. Many of the then newly appearing medical disciplines defined their fields around particular diseases and organs. Thus, in many cases, a rather isolated understanding of particular diseases came at the cost of more ‘holistic’ understandings of the human body [20].

In this regard, cardiology seems to be a paradigmatic case. Certainly, the heart is cardiology’s principal research object, legitimating the discipline’s very existence. In this article, we argue that, since its inception at the beginning of the twentieth century, cardiology has been continuously expanding, i.e., increasing the scope of its events of comparison. In particular, there were three surges, which rapidly pushed the discipline towards global significance: the foundational years (from approximately 1900 to 1950), the post-war era (from approximately 1950 to 1980), and the neoliberal era (from 1980 until today).

According to Heintz and Werron’s theoretical model, a precondition for globalisation processes to set in consists of the continuous production of comparable events. So, what does that mean for the discipline of cardiology? Here, we want to single out four factors that have contributed to make cardiological research and knowledge the content of comparisons beyond the border of nation states. These factors have largely interacted with one another, and they relate both to globalisation through linkages and connectivity, as well as to globalisation through description. As these factors have been contributing to the establishment of a global horizon of cardiological comparisons, they have also been crucial for bringing ever more attention to particular cardiovascular health risks, and thereby connecting cardiology to the arena of global public health.

### 4.1. Technological Innovations Helped Cardiological Objectivity Take Shape and Institutionalise the Discipline

Technological devices that made the heart and blood circulation measurable epistemic objects are the first important factor. They contributed to what can be called the laboratorisation of cardiology, and the emergence of a specific objectivity [21] of cardiology’s most important research objects, namely the heart and the blood circulation. The perfecting of the microscope in the nineteenth century enabled important progresses in particular medical fields, such as anatomical pathology and bacteriology, but also enormously shaped medicine on the whole [1]. For the formation of cardiology, the inventions of such devices as the sphygmomanometer and the stethoscope had certainly been important steps, but it was the introduction of the electrocardiogram (ECG) that contributed the most to institutionalise methods that could produce objective, and thereby comparable, data on the heart and blood circulation. The ECG also seems to have been a pivotal technology that made the institutionalisation of cardiology, as a discipline, possible at all. Already around 1900, the Dutch engineer Willem Einthoven had constructed a first version of a string galvanometer to measure the electric currents produced by the heart. Einthoven was later awarded a Nobel Prize for this discovery, but it took Einthoven’s collaboration with the British physician Thomas Lewis to popularise the technology amongst cardiologists around the world. Based on the data produced with his fist ECGs, Einthoven began to look for correlations between the patterns of the heart’s electric currents and specific cardiac symptoms. Moreover, Einthoven was a skilful mediator between, on the one hand, the worlds and languages of physicians, and, on the other hand, that of manufacturers and technicians [22]. Lewis, in turn, who had been in close correspondence with Einthoven since 1909, was one of the first clinical researchers who had begun to use the ECG to study cardiac conditions. For his famous ‘Soldier’s Heart’, he produced more than 10,000 ECG images of soldiers who had fought in World War I. He also published one of the first textbooks in cardiology, which included visual depictions of the heartbeat and helped to interpret data produced by the ECG [23]. Thus, in collaboration with Lewis, Einthoven was able to bring the ECG out of the laboratory, into mass production, and to eventually establish the ECG as the standard diagnostic device of cardiology, which it still is today.

In sum, without putting the case for technological determinism, it is safe to say that certain technological innovations, in particular the invention and subsequent mass production of electrocardiographic machines, allowed the heart and the blood circulation to emerge as unitary epistemic objects, which then served cardiologists and others as a basis for communicating comparisons (of research) all over the world. These technologies helped cardiology emerge as a discipline in its own right, on the level of national health systems, but also facilitated the discipline’s globalisation.

### 4.2. Newly Emerging Cardiac Societies Created a Worldwide Institutional Underpinning

Partly as an immediate consequence of the establishment of the heart and blood circulation as genuine epistemic objects, the emergence of cardiology as a discipline separate from internal medicine contributed to making cardiological knowledge and research comparable across countries. However, another factor that helped establish the communication of comparisons beyond national borders was that cardiology had been institutionalised in a large number of countries. The appearance of new national cardiac societies is a good indicator of this tendency of expansion.

**Figure 1:**
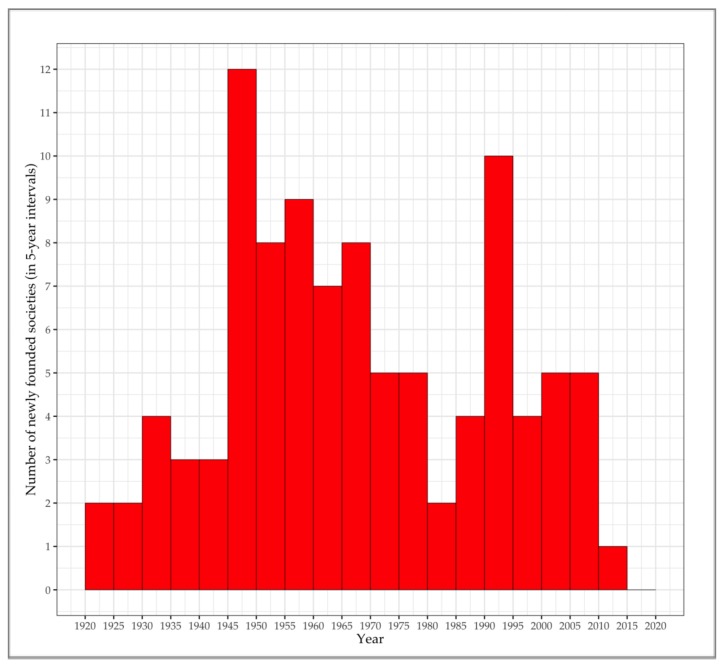
New foundations of national cardiac societies between 1920 and 2015 (n = 99). Source: authors’ own compilation of data retrieved from the webpage of the ESC [25] and others.

Figure 1 shows how national cardiac societies have been founded over approximately the last century, with the last organisation included in the dataset having been founded in 2012. There were two waves of foundations. The first wave began with a number of pioneering cardiac organisations, and peaked in the five years following World War II. These were predominantly organisations from North America, Europe, and later from South America, as shown in Table 1. The second wave of foundations (not shown in Table 1) seems to be related to the disintegration of the Soviet Union, as most of the national cardiac organisations founded after 1990 are from countries of the former Soviet bloc.

From a geographical point of view, cardiology started to institutionalise from two regions: America and Europe. Almost all of the national cardiac organisations appearing in the foundational years were from these regions. Geographical and cultural proximity seem to have been important factors, establishing new transnational linkages, and thereby spreading the idea of national cardiac societies throughout these regions. It is tempting to see a ‘contagion’ effect at work here. Indeed, according to Reichert [24], the incorporation of the American Heart Association can be understood as a consequence of the physician Louis Bishop’s visit to London, where he was urged by the cardiologist James Mackenzie to establish cardiology in the United States. Likewise, the foundation of the German national cardiac society seems to have had an effect on new foundations in countries neighbouring Germany. On the other side of the Atlantic, aside from the United States, several Central and South American countries founded national cardiac societies in the 1930s and 1940s, eventually even leading to the incorporation of the first regional cardiac organisation, the Inter-American Society of Cardiology, in 1944.

That is, with the exception of the ‘geographic outliers’ of Japan and India, in the foundational years of global cardiology, new national cardiac organisations were incorporated exclusively in Europe and America.

In addition to national cardiac societies, newly emerging regional organisations have contributed to expand the institutional foundations of global cardiology. Concomitantly with the first World Congress of Cardiology, the European Society of Cardiology was incorporated in 1950. Other regional professional organisations, such as the ASEAN Federation of Cardiology and the Inter-American Society of Cardiology, either followed suit, or had come into existence at the same time or even earlier. In 1978, with the creation of the World Heart Federation, cardiology even received its official global representative organisation. In sum, over the post-war era, cardiology’s institutional underpinning had been put on a stable footing. 

### 4.3. Cardiological Journals Proliferated and Provided a Communicative Infrastructure

With the institutionalisation of cardiology in a range of health care systems over the world, the emergence of medical journals specifically dedicated to cardiology followed suit. Some of today’s leading cardiological journals, including Circulation and Circulation Research, were established either already during the foundational years, or in the post-war era. In the first half of the twentieth century, cardiological journals newly appearing on the scene mostly took a general outlook. In the second half of the century, however, more and more journals dedicated to specialist fields of cardiology appeared.

**Figure 2:**
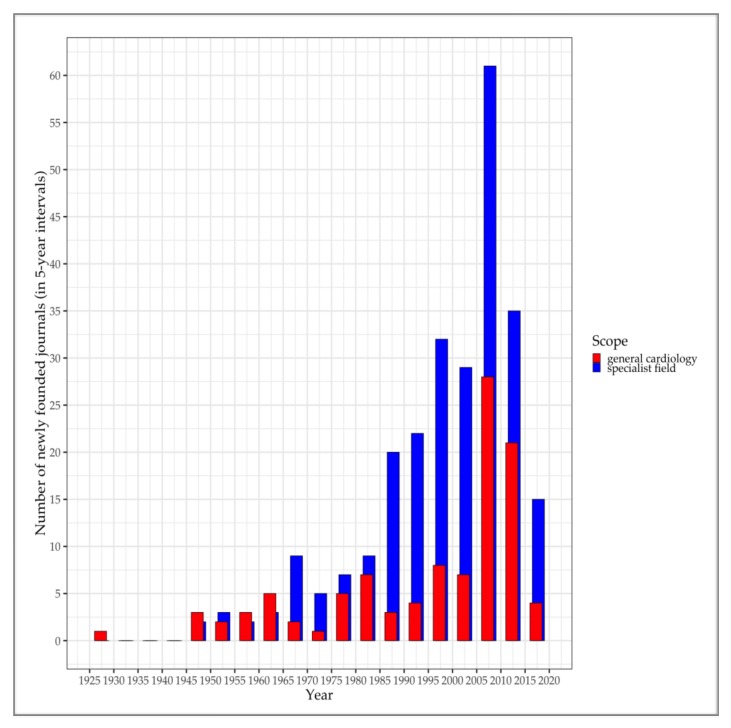
New foundations of cardiological journals between 1925 and 2018. Source: authors’ own compilation based on dataset from Scimago Journal and Country Rank [26].

Our analysis of the SCImago dataset revealed that 104 of 358 journals have a general cardiological scope. The remaining 254 journals have a specialist scope, i.e., they predominantly feature articles on a specialist field of cardiology. Figure 2 shows the increase in the number of foundations of ‘general’ and ‘specialist’ journals, summed up to 5-year intervals (note that data for 2018 and 2019 were not yet available at the time of the writing of this article). On the one hand, there is a clear tendency for more and more journals to appear on the scene, with large increases over approximately the past four decades. On the other hand, Figure 2 shows that specialist journals have contributed the major shares to increases in new foundations since 1965. Moreover, since 1985, the number of newly incorporated specialist journals have almost continuously been more than twice the number of foundations of general cardiology journals.

These trends evidence an ever more specialised and differentiated cardiological field. It is certainly true that specialisation and differentiation set in already in the years following World War II, but these processes seem to have really been boosted over the last four decades or so. As we will show in the next section, this is exactly the timespan during which conditions for the globalisation of heart failure research have been installed and eventually allowed the subspeciality to globalise. We may assume that this has been a rather general trend, and that many other subspecialties of cardiology have emerged around the same time. 

As in the case of foundations of national cardiac societies, a more detailed look into the foundational years of the discipline facilitates our understanding of how cardiology has become global. Table 2 shows all journals whose first year of coverage was before 1980 and whose scope is general cardiology. As can be inferred in some cases from the title or language of the journal, many of them were founded on behalf of national cardiac organisations, such as the American Heart Association, or the Indian, Chinese, Russian, and Polish cardiac societies. The emergence of medical journals that have been connected with, and often been published on behalf of, national cardiac organisations all over the world may itself be interpreted as a process of globalisation. And it is definitely consistent with the neoinstitutional assumption that global scripts have been emulated across the world by nation states and other actors, resulting in increasingly similar institutional structures. However, another important implication is that the nearly worldwide institutionalisation of cardiological journals has brought about a communicative infrastructure that could be utilised by cardiologists from around the world to put their own research into a global reference framework. That is, the worldwide net of general and specialist cardiological journals has facilitated the communication of comparisons. Although horizons of comparison have initially been constructed on a national and regional scale, a global horizon of comparison has been emerging throughout the second half of the twentieth century.

### 4.4. Cardiology and Its Professional Knowledge Became Increasingly Standardised and Comparable

Since the discipline’s inception, cardiological knowledge has increasingly become standardised internationally. This trend can be observed in different domains, e.g., the standardisation of cardiological language, the standardisation of methods and units of measurement, and others. Moreover, the increasing standardisation of cardiology has led to more and more quantifiable and comparable data repositories. As we show in this subsection, this resulted not only in the further stabilisation of a global horizon of cardiological comparisons, but also facilitated the emergence of particular CVDs on the agendas of global public health. 

Arguably, the introduction of the ‘International Classification of Diseases’ (ICD) has been one of the most important drivers for standardisation of health on a global scale [27]. The ICD originated from efforts around the International Statistics Institute to make mortality rates comparable across countries, at the end of the nineteenth century. The Institute’s classification itself originated from a system of mortality classification that had for long been in use in the city of Paris. In the 1920s, the newly founded Health Organisation of the League of Nations began to show interest in the International Statistics Institute’s mortality statistics. Moreover, the organisation made efforts to develop a standardised classification system for collecting comparable morbidity rates across countries. Although they included, at the maximum, data from thirty-seven, mostly European countries, these morbidity statistics were the first attempt to make health statistics internationally and comprehensively comparable [28].

At the World Health Assembly in 1948, however, a completely new and more complex classification system for the causes of morbidity was prepared and introduced. This sixth version of the ICD laid the foundation for a number of new cardiological statistics, some of which were introduced in the following years. Nevertheless, in the 1950s, epidemiologists still faced enormous difficulties in developing methods that would allow cross-national comparability, as different countries were still using classifications systems that partly differed considerably amongst countries. These problems were only alleviated in the 1960s, when the next revised version of the ICD was published [28].

However, in 1958, an expert committee of the World Health Organisation (WHO) had already convened in Geneva to discuss problems with the standardisation of the diagnoses of hypertension and coronary heart disease. The committee was exclusively concerned with hypertension and coronary heart disease, because these diseases had previously been defined as the major causes of cardiovascular deaths. The WHO planned to conduct cross-national epidemiological studies that would allow generalisations at a global scale. Standardisation was deemed a necessary first step on this way. Internationally valid standards were necessary, before epidemiological studies with a global outreach could be conducted [29]. The WHO’s 1959 report thus recommended to set in motion long-term changes on the level of all member states by standardising the educational curricula of cardiological professionals, and by establishing epidemiological studies under standardised laboratory conditions [29]. Although we have not yet investigated this question, it is likely that these initiatives have led to a considerable degree of worldwide convergence of professional training in cardiology. Consequently, today professional cardiologists are familiar with techniques, technologies, practices, and nomenclature, that are widely similar around the world, allowing, for instance, health workers in cardiology to take along their professional stocks of knowledge as an asset, when they decide to migrate to another country [30]. This kind of transnational transfer of professional knowledge would hardly be possible without the foregoing extensive international standardisation of cardiology.

Moreover, from the 1950s onwards, apart from the WHO, other international organisations, such as the International Labour Organisation, the Organisation for Economic Co-operation and Development, or the United Nations Development Programme, had begun to develop more sophisticated and at the same time more standardised methods for measuring the financing of health care systems and their performance. According to Gorsky and Sirrs [28], the 1980s in particular witnessed efforts to measure health from an economic perspective, a trend that reached its peak with the World Health Report 2000. This document perfected the ranking of country’s health systems, according to a range of internationally standardised indicators. Prior to that, the Global Burden of Disease (GBD) [31], a report compiled by researchers from the WHO and the Harvard School of Public Health, had made a first comprehensive attempt to compare the ‘burden of diseases’ across countries, and thereby make the accumulated burden visible at a global scale. This report introduced the new metric of DALYs (disability-adjusted life years), which made it possible to relate the health of a country’s population to its economic performance. Not surprisingly, the World Bank picked up the concept, and featured it in its 1993 World Development Report [32].

Interestingly, the GBD was also pioneering another new perspective, as it payed much more attention to NCDs than had be done in the preceding decades, so that amongst them a number of CVDs, in particular ischaemic heart disease and stroke, emerged as issues of global health concern. Since then, an understanding has arisen in global health circles that CVDs represent major global health burdens. Moreover, the aforementioned reports facilitated the communication of new comparisons regarding diseases. For instance, it is now common to compare the burden of individual CVDs or CVDs as a disease group with other diseases and disease groups, within countries and across countries.

In sum, there has certainly been ever more international standardisation of cardiology over a wide range of dimensions. Identical standards across many countries of the world are a precondition for producing cross-national comparisons. With the emergence of a global communicative infrastructure and a global horizon of comparison that scaffold the continuous communication of comparisons, global cardiology has also created platforms to influence the policies of global health.

## 5. How Heart Failure Went Global

In the previous section, we demonstrated how cardiology has been continuously expanding and transcending its initially national and regional boundaries, and then growing into a truly global discipline over the last century. We also argued that the communicative infrastructure and the global horizon of comparison that emerged accordingly, offered new possibilities to relate cardiovascular diseases to the domain of global health policies. In this section, we argue that these possibilities can be further enhanced in such cases, where a medical discipline’s subspecialities are emerging, which are concerned with specific diseases or health risks. In order to illustrate our argument with regard to the case of cardiology, we take a closer look at the clinical syndrome of heart failure, and how this particular cardiological subspeciality has emerged in recent decades.

### 5.1. New Clinical Trials and Treatment Strategies Drove the Institutionalisation of Heart Failure

Heart failure is not an entirely new domain of cardiology. For instance, Riegger [33] notes that heart failure had a prominent place in lectures given at the inaugural session of the German cardiac society in 1928. However, many national cardiac societies began to establish special sections, associations, or working groups, dedicated to the syndrome, only in the early 1990s. This trend has continued over the last three decades, and ever more national cardiac organisations created such working groups and special interest groups. Moreover, two important organisations with a scope beyond national borders were created in the 1990s: the ESC’s Heart Failure Association and the Heart Failure Society of America [34]. 

It is unsurprising, then, that heart failure research began to develop a stable institutional grounding around that time. According to Riegger, in the late 1980s, a number of clinical trials had revolutionised the treatment of heart failure [33]. Specifically, the results of the CONSENSUS trial, published in 1987, had demonstrated the role of ACE-inhibitors to significantly improve outcomes for heart failure patients. Importantly, it had also shown that and how the treatment of heart failure could be put on the footing of so-called evidence-based medicine and its accompanying methods, in particular, randomised controlled trials [35]. Moreover, according to Katz and Konstam [36], the very definition of heart failure prevailing today, which pays attention to the causes of the heart muscle’s deterioration, was also derived around the same time. 

Concomitantly, with the establishment of special interest groups within national and regional cardiac societies, the firmer institutionalisation of heart failure (research) spread to other areas as well: universities created chairs in cardiology with special focuses on heart failure; research networks were established, such as the German Competence Network for Heart Failure [37], along with advocacy and policy organisations, e.g., the Heart Failure Policy Network [38]; and online resources that cover the topic from multifaceted perspectives have mushroomed.

The institutionalisation of heart failure groups, societies, and associations, the appearance of heart failure as a special interest of cardiology chairs, and ever more clinical studies defining heart failure as a field for clinical research, are clear indications of the further differentiation of this subspeciality from cardiology. The new waves of clinical research in this emerging subspecialty, but also, heart failure as a topic of concern in areas outside of clinical research, seem to have been necessary preconditions to create a stable basis for the continuous production of new knowledge about the syndrome, at an increasingly global scale.

These developments thus stabilised the institutional underpinnings that allowed the continuous production of comparable events in the field, in terms of new research, new studies, which are eventually communicated at conferences, in books, and articles. We will now have a closer look at how the research landscape of heart failure has developed over the last three decades in terms of publication output.

### 5.2. Specialist Journals and Guidelines Provided Means for Communicating Research at a Global Scale

Apart from the institutionalisation of a new research infrastructure, another indication that heart failure has become an important subspeciality of cardiology is the appearance of specialised literature on the topic, including textbooks and handbooks, journals, and also clinical practice guidelines. As for handbooks, Katz and Konstam already demonstrated how the emphasis on treatment options has changed towards the now prevailing paradigm of heart failure treatment, which has included ever more effective drugs [36]. Additionally, more and more textbooks, handbooks, and atlases exclusively dedicated to heart failure have appeared over the last decades.

However, as was already noted by Ludwik Fleck in 1935 [39], scientific journals are the real arbiters deciding on a discipline’s current state of knowledge. Newly created medical journals dedicated to heart failure are thus an important indicator for the growing significance of the subspeciality. Table 3 shows the journals from the SCImago-based dataset that are specifically dedicated to heart failure. Consistent with our initial observations in regard to the increasingly specialisation of cardiological journals, heart failure journals have been founded since the middle of the 1990s, i.e., at a time when national cardiac societies started creating heart failure working groups and associations, and when new effective treatment options were becoming available. Table 3 also reveals that many of the new heart failure journals have a huge impact, measured by the SCImago journal rank. Five of the nine journals occupy ranks in the upper quartile of all 358 cardiological journals. This indicates the subspeciality’s enormous significance within cardiology. Interestingly, four of these journals are also associated with, or published on behalf of, members of an informal coalition of organisations that we dubbed the ‘transatlantic five’ of heart failure elsewhere. We argued that the worldwide knowledge production in regard to heart failure is largely structured in terms of a logic of epistemic centres and peripheries, with the transatlantic five (ESC, HFA, ACC, AHA, and HFSA) being the dominating actors of the centre [34].

**Figure 3:**
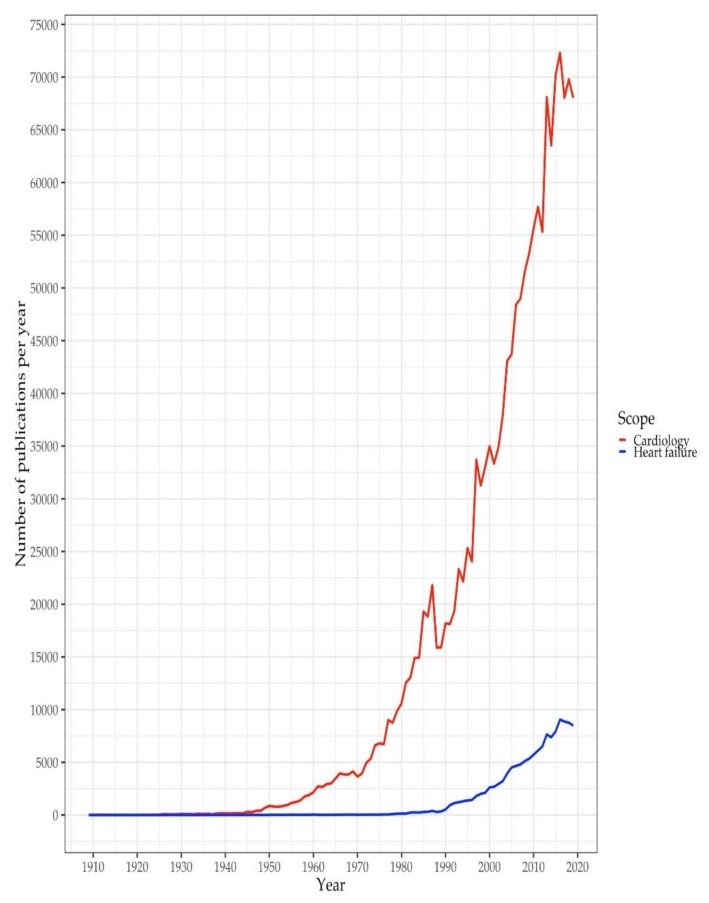
Publication output in cardiology and heart failure between 1910 and 2019 (based on a dataset compiled from Web of Science data).

Taken in its own right, the fact that some new journals have been recently introduced to the cardiological community—though in part, they even rank high according to citation impact—is but one indication for the growing significance of heart failure as a genuine subspeciality. However, this assumption is additionally supported by the subspeciality’s publication output in relation to cardiology in general. Figure 3 shows the development of publication output in the field of cardiology in general, and on the topic of heart failure in particular. What is of interest here is not so much the enormous increase in publications in absolute numbers, but rather, how the publication output on heart failure has increased in relation to the publication output in the whole field of cardiology: from 1979 onwards, more than one percent of the total annual publications in cardiology have continuously been on the topic of heart failure; from 1991 onwards, of all annual publications in cardiology, even more than five percent have been on the topic of heart failure. This trend reached its preliminary peak in 2017, with approximately thirteen percent of all cardiological research published in this year being on the topic of heart failure.

The increasingly large share of research on heart failure over the past four decades is consistent with the enormous growth in specialist cardiological journals over the same period. Of course, the sheer existence of more specialist journals has simply allowed to publish more specialist research. However, amongst the diverse specialist research fields and subspecialties, heart failure seems to occupy a particularly salient position. Although we have not yet directly compared the publication output on heart failure research with that in other fields and subspecialties, it seems safe to say that the subspecialty of heart failure has experienced particular growth. Its special position within cardiology is also reflected in the central position that clinical practice guidelines on heart failure have had over the last three decades. Guidelines on heart failure have been amongst the most frequently cited documents in cardiology. Our search on Web of Science revealed that some of the most frequently cited documents in the research field that Web of Science refers to as ‘Cardiovascular System & Cardiology’ are recommendations, classifications, scientific statements, and guidelines concerning heart failure. Among a total of 1,603,129 documents in this research field, the guidelines on heart failure published by the European Society of Cardiology in 2016 [40], and the guidelines published by the American Heart Association/American College of Cardiology/Heart Failure Society of America in 2013 [41] rank 15th and 25th, respectively. Moreover, our previous analysis [34] demonstrated that there has been a trend in the global cardiological community to develop clinical practice guidelines for the diagnosis and treatment of heart failure since the 1990s, and that this trend has spread to many countries and cardiac organisations around the world.

In sum, the worldwide communicative infrastructure of cardiological journals that had been installed in the second half of the twentieth century, has been piggybacked by the subspeciality of heart failure over approximately the past three to four decades. This newly emerging communicative infrastructure, in turn, allowed the subspeciality to put its now continuously appearing research to be put into a global horizon of comparison.

### 5.3. Heart Failure Nomenclature and Standards Globalised

While the previous subsections demonstrated how the institutional underpinnings of the continuous production and communication of new research on heart failure have been developed over approximately the last three decades, we have not yet examined the specific conditions that make research on heart failure comparable beyond particular local contexts. We argue that an increasingly standardised nomenclature has been established at a global scale, allowing research done in a particular locality to be understood in relation to research done in any other locality of the world. Since this nomenclature is complex and highly technical, in order to make our case, we briefly pick out only one instance of global standardisation here.

Natriuretic peptides (NPs) are a good case in point, because their clinical introduction, according to Mueller [42] ‘constitutes the most important advance in the diagnosis of heart failure in the last decade’. These peptides induce diuresis and natriuresis, i.e., the excretion of urine and sodium from the kidneys. There are several known subtypes. One of them, brain type natriuretic peptide (BNP), together with its precursor, NT-proBNP, is now routinely, and in widely standardised ways, used for diagnostic and therapeutic purposes all over the globe. More recently, NPs are even being discussed as a candidate biomarker for a universal definition of heart failure [43]. We may ask how they have obtained this role.

A series of papers published in the early to mid-1980s, starting with a publication by a research team around Adolfo J. de Bold from a Canadian laboratory, demonstrated the existence of hormones that were produced primarily in the myocardium. Before this discovery, there were some speculations about the possibility of an endocrine function of the heart, i.e., the fact that hormones are produced in the heart, but no actual prove did exist. De Bold’s team showed that injecting extracts from the atrium into rats caused increased diuresis, natriuresis and also reduced blood pressure [44]. Accordingly, they spoke of an atrium natriuretic factor (ANF) then. After de Bold’s initial experiments, a number of research groups and laboratories undertook further studies. Oikawa et al. [45] managed to isolate a peptide, which they referred to as atrium natriuretic peptide (ANP). They were able to confirm the causal relations that de Bold’s group had found. Subsequently, in 1988, biochemists from the Miyazaki Medical College (Japan) published a paper on their discovery of a novel peptide they had found in porcine brains [46]. Parallel to laboratory research on ANP, Sudoh’s group injected synthesised BNP into assay rats and found similar effects like in ANP, i.e., reduced blood pressure, natriuresis, and diuresis. But they also found genetic differences from ANP, and therefore deemed it to be a separate natriuretic peptide. As a result of the fact that they discovered this peptide in the animals’ brains, they referred to it as brain natriuretic peptides (BNP). However, later, the heart’s ventricles were identified as the primary production sites of BNP.

Throughout this discovery process, natriuretic peptides have emerged as a stabilised ‘scientific object’, in the sense discussed by Rheinberger [18], or a rich model of theorisation, as discussed by Strang and Meyer [47]. That is, today, the causal relations between cardiac NPs and the cardiovascular system have become pretty clear: the net effect of the properties of ANP and BNP ‘is balanced vasodilatation in the arterial and venous beds as well as natriuresis and diuresis’ [48].

As Strang and Meyer [47] have argued, the theorisation and the building of abstract models tend to facilitate processes of diffusion. The detailed unearthing of the objective properties of NPs, making them a ‘scientific object’ and a complex cardiological model, seems to have been a precondition for the creation of new opportunities for further studies, and also for the use of this object in clinical practice, and both of these considerations apply on a new global scale. First, natriuretic peptides have increasingly been employed in the context of clinical studies (often randomised controlled trials). That is, BNP, and by implication NT-proBNP, now often constitute parts of technological research environments. For instance, in PARADIGM-HF [49], a randomised controlled trial comparing a novel drug (LCZ696) against the established ACE-inhibitor Enalapril, levels of BNP / NT-proBNP in patients’ blood plasma were used as a criterion for including patients into the study population. Aiming for conclusions with a scope as general as possible, i.e., with a global scope, PARADIGM-HF included subjects from a number of continents and regions. In effect, the new heart failure drug that is now being produced and put onto the market by Novartis in more than 120 countries of the world, is also a highly standardised drug, with a potentially global scope.

Second, concomitantly with NPs having become a regular technological object in clinical studies, they have also become a clinical object, in the sense that they are ‘a tool with which to determine whether a patient has congestive heart failure and to measure its severity’ [50]. Therefore, NPs have also made their way into clinical practice guidelines for the diagnosis and treatment of heart failure. As we previously demonstrated [34], recommendations with regard to the use of NPs as diagnostic tools have become exceedingly similar across a number of heart failure guidelines from a range of different countries.

In sum, NPs are but one example for international standardisation in cardiology with particular significance for the subspeciality of heart failure research. They are used as a standardised measurement tool to decide on including patients in study cohorts in big international clinical trials, in which translocal generality and comparability are necessary cornerstones; they are recommended for diagnostic purposes in current clinical practice guidelines, in highly standardised terms across a range of guidelines from different countries; and they are used as a clinical tool (a biomarker) in diagnosing heart failure in health care systems around the world, thereby also making available cross-country comparative statistics. Thus, while the construction of NPs as a scientific object and a theoretical model itself relied on the possibility of global comparison, NPs have provided an important means to produce comparisons, communicate, and relate them to global reference frames.

## 6. Discussion

This research connected with neoinstitutionalist theories on globalisation and world–society, specifically with Heinz and Werron’s approach to study the preconditions of globalisation [13]. It reconstructed how a global rationalised field, the field of cardiology, has emerged. A special emphasis has been given to heart failure as a clinical syndrome and a subspeciality of cardiology. Through our theory-guided empirical survey, we come to the conclusion that both cardiology and heart failure have increasingly globalised against the backdrop of international and transnational interdependence, not only in economic, financial, social, and political terms, but primarily in medical terms. Biomedical globalisation incorporates the simultaneous globalisation of various functionally differentiated sub-spheres and subfields of an emerging global system of medicine, including communicable diseases and non-communicable diseases. The perception of diseases and epidemics as common risks for humanity in the world–society, the universal declaration of human rights, and the proclamation of health rights as fundamental human rights for all citizens [51], along with the global diffusion of evidence-based biomedicine, and finally the global diffusion of medical technological devices have relativised the relevance of national borders and facilitated the globalisation of cardiology and heart failure.

In this article, we have engaged with cardiology and heart failure from mainly two perspectives of globalisation research, namely with a focus on globalisation through linkages (of actors and institutions), and with a focus on globalisation through description (as communicated comparisons). Both of these perspectives on—and, indeed, mechanisms of—globalisation can be understood as two sides of a coin, as they have complemented each other, and only through their interaction have the phenomena that we were interested in been constituted.

We have identified a number of preconditions that had to be met before cardiology—and later heart failure research—could globalise. At the beginning of the twentieth century, technological innovations, specifically the ECG, turned the heart into an epistemic object around which an entire medical subdiscipline could be built. It also provided cardiology with a very specific sense of objectivity, so that, since the invention of the ECGs, ECG images of the heart have served as a medium allowing clinical researchers to publish their findings within a standardised framework, and to relate their own research to those of others using the same technology. In other words, technologies such as the ECG have fostered comparisons, which have been an important mode of the globalisation of cardiology. Moreover, with the examples of national and supranational cardiac societies, along with the growth of medical journals, we have demonstrated how cardiology continuously expanded over the twentieth century. The creation of this worldwide institutional underpinning has pushed forward the emergence of global horizons of cardiological comparison, further contributing to the globalisation of the discipline itself. Finally, the worldwide standardisation of cardiological knowledge has also stabilised comparability. All of these observations support the assumption that, in particular, three preconditions must be met such that globalisation in a medical field can set in: a solid basis for the production of comparable events, the possibility of comparisons beyond local contexts, and an overarching framework linking individual events of comparison. Our analysis of heart failure research has shown that these preconditions can be analytically applied in a similar manner to medical subspeciality fields.

With our investigation of the emergence of cardiology as a global discipline, and the differentiation of heart failure research in more recent decades, we are in a position to dare some tentative conclusions as to the question of whether globalisation of one particular societal field necessarily implies the globalisation of its subfields. At first, this question must probably be answered in the negative: it is difficult to imagine any social mechanism that could make the globalisation of a cardiological subspeciality a necessary consequence of its initially local appearance. In fact, however, heart failure research—certainly along with many other cardiological subspecialties—did globalise, against the backdrop of an already global field of cardiology. This empirical observation, then, points to the possibility of particular institutional mechanisms and conditions that increase a societal subfield’s likelihood to globalise under the condition that its ‘parent’ field is already global. This certainly includes an existing infrastructure of organisations, professional actors, scientific journals, and, of course, a global horizon of comparison—components that we have described in this article.

The globalisation of an overarching societal field and its subfields rarely ever proceeds in a completely congruent manner. In our case study, we demonstrated that, while they have been globalising, the fields of cardiology and heart failure each had have their own autonomous momentum and dynamics, and even the timing and tempo of their globalisation has differed. In consequence, there has been a delaying effect with regard to each of these fields’ globalisation: in the field of heart failure research, globalisation really began to gain momentum in the 1990s, approximately half a century after the globalisation of cardiology as a whole had set in. However, the subspeciality has globalised with enormous speed in the past three decades, and is about to achieve the same global reach as cardiology in the world–society. Here, we have only touched on the phenomenon of (in-)congruencies of societal fields’ and their subfields’ processes of globalisation. That is a topic that certainly deserves more academic attention, and future research is needed.

## 7. Conclusions

Of course, the approach taken to the study of globalisation in this article could itself be complemented by other perspectives. For example, in the hope of gaining further insights into the relevance of social actors in relation to the linkages, institutions, and communicative infrastructures that we have described, we could give our conceptualisation a slightly different twist and build it around the following four dimensions. 

Firstly, from the perspective of actor-constellations, we have demonstrated the fast growth in the number of cardiological associations and societies at the national level. This growth was initiated in the interwar period, and has accelerated in development, and thrived in the post-war epoch throughout the rest of the twentieth century. Some macro-regional cardiological societies, from Europe and the United States, have in fact taken the global lead in promoting universalistic scripts on the diagnosis, treatment, and rehabilitation of cardiovascular diseases and, in particular, of heart failure. We propose the concept of the ‘transatlantic five’ to identify the most important and influential cardiac actors that have been taking a dominating role in generating global discourses and standards. This includes the ESC/HFA from Europe, and the ACC/AHA/HFSA from the USA. A transatlantic bipolar power constellation has been prevailing in the global knowledge market on cardiovascular diseases and heart failure. At the formal and symbolic level, the World Heart Federation represents a huge number of national cardiac organisations around the world.

Secondly, from a diachronic perspective, we have shown that the global discipline of cardiology has been incrementally established, i.e., through a number of stages: in the foundational years from 1900 to 1950, with a still limited reach in geographical terms; then, in the post-war era, a number of international agencies and cardiological organisations were gaining more relevance, contributing to the forging of a global organisational architecture, and creating new networks for cardiovascular diseases around the globe; and in recent decades, there has been a new wave of foundations of national cardiac organisations, and cardiology has become an even more specialised and differentiated discipline.

Thirdly, in terms of a spatial and organisational perspective, several supranational organisations have extended their activities into different world regions, including regional cardiac societies and associations in nearly all continents, such as the PASCAR in Africa, the APSC in the Asia-Pacific, the SIAC in the Americas, and the PACHDA in the Near East. That is, many national cardiac societies have joined together and become part of supranational cardiac organisations.

Fourthly, in terms of a social dimension, we have reconstructed how a network of highly professional and recursive communication has emerged through academic journals, publications, discourses and scientific citations in the field of cardiovascular diseases and heart failure. We demonstrated that academic journals for general cardiology and specialised fields have mushroomed over the last three to four decades.

Benefiting from the supranational and international organisational setting, the global scripts in the arena of cardiovascular diseases and heart failure have spread worldwide, contributing to global benchmarks for standard-setting and standard-application in these (sub-)fields. A repertoire of semantics, discourses, models, and metrics, along with standardised diagnostic and treatment procedures has been disseminated worldwide. The periodical adoption of guidelines on heart failure developed by the ESC/HFA and the ACC/AHA/HFSA, and their constant updating, have, in fact, resulted in the worldwide diffusion of standards on the treatment of cardiovascular diseases and heart failure. This, in turn, results in simultaneous and similar perceptions with regard to medical state-of-the-art knowledge and novel clinical research results around the world. In other words, an ecumenical context of observation, perception, and comparison has emerged in the arena of cardiology, and particularly in the field of heart failure, incorporating nation states and national cardiac societies into a comparable biomedical framework at a world scale.

A corollary observation with regard to the emergence of that kind of horizon of comparison, which we only touched on in this article, is that through the quantification of medical fields and the provision of rationalised data-structures and metrics, e.g., in regard to the mortality and morbidity rates of particular diseases, by international and supranational organisations, all countries, those of the Global North as well as those of the Global South, can be compared with each other in the field of cardiovascular diseases. The thriving world events in the global arena of cardiology, including international symposiums, conferences, congresses, and annual meetings, driven by some de facto global actors, such as ESC, ACC/AHA, and WHF, have further contributed to a cosmopolitan interaction and communication arena beyond the scope of national and local medical communication. It seems that, since the last quarter of the twentieth century, the national cardiological communities have become increasingly detached from local medical experiences, and have become ever more integrated into a macro- and global communication context. This huge and complex institutional infrastructure has served as the backdrop from which particular cardiovascular diseases, and the syndrome of heart failure, could easily emerge as topics of global concern.

## Figures and Tables

**Table 1:** New foundations of national cardiac societies before 1950.

National Cardiac Society	Year of Foundation
British Cardiovascular Society	1922
American Heart Association	1924
German Cardiac Society	1927
Czech Society of Cardiology	1929
Netherlands Society of Cardiology	1934
Belgian Society of Cardiology	1935
Japanese Circulation Society	1935
Mexican Society of Cardiology	1935
French Society of Cardiology	1937
Argentine Society of Cardiology	1937
Cuban Society of Cardiology	1937
Brazilian Society of Cardiology	1943
Colombian Society of Cardiology and Cardiovascular Surgery	1943
Spanish Society of Cardiology	1944
Canadian Cardiovascular Society	1946
Romanian Society of Cardiology	1947
Swedish Society of Cardiology	1947
Peruvian Society of Cardiology	1947
Hellenic Society of Cardiology	1948
Swiss Society of Cardiology	1948
Chilean Society of Cardiology and Cardiovascular Surgery	1948
Cardiological Society of India	1948
Uruguayan Society of Cardiology	1948
Irish Cardiac Society	1949
Portuguese Society of Cardiology	1949
American College of Cardiology	1949

Source: authors’ own compilation of data retrieved from the webpage of the ESC [25] and others.

**Table 2:** New foundations of cardiological journals before 1980.

Journal	First Year of Coverage	SJR Rank
American Heart Journal	1925	24
Acta Cardiologica	1946	220
Circulation	1950	2
Arquivos Brasileiros de Cardiologia	1950	210
Circulation Research	1952	5
Kardiologia Polska	1954	213
Revista Argentina de Cardiologia	1957	299
Progress in Cardiovascular Diseases	1958	22
American Journal of Cardiology	1958	43
Revista Espanola de Cardiologia	1961	170
Indian Heart Journal	1961	209
Cor et Vasa	1961	272
Kardiologiya	1961	289
Cardiology	1964	108
Cardiovascular Research	1967	33
Scandinavian Cardiovascular Journal	1967	172
Basic Research in Cardiology	1973	50
Current Problems in Cardiology	1976	54
Clinical Cardiology	1978	74
Herz	1978	223
Chinese Journal of Cardiology	1979	286

Source: modified dataset retrieved from Scimago Journal and Country Rank [26].

**Table 3:** New foundations of heart failure journals.

Journal	First Year of Coverage	Related to/Official Journal of	SJR Rank
Journal of Cardiac Failure	1994	HFSA	45
Heart Failure Reviews	1996		48
European Journal of Heart Failure	1999	ESC/HFA	6
Current Heart Failure Reports	2004		116
Heart Failure Clinics	2005		151
Circulation. Heart failure	2008	AHA	11
Insuficiencia Cardiaca	2010		321
ACC: Heart Failure	2013	ACC	9
ESC heart failure	2017	ESC/HFA	138

Source: modified dataset retrieved from Scimago Journal and Country Rank [26].
